# ACORN SDOH survey: Terminological representation for use with NLP and CDS

**DOI:** 10.1017/cts.2024.24

**Published:** 2024-02-06

**Authors:** Melissa P. Resnick, Diane Montella, Steven H. Brown, Peter Elkin

**Affiliations:** 1 Department of Biomedical Informatics, University at Buffalo, Buffalo, NY, USA; 2 U.S. Department of Veteran Affairs, WNY VA, Buffalo, NY, USA; 3 U.S. Department of Veteran Affairs, Office of Health Informatics, Washington, DC, USA; 4 Faculty of Engineering, University of Southern Denmark, Odense, Denmark

**Keywords:** Biomedical informatics, clinical care, social determinants of health, ACORN survey

## Abstract

**Objective:**

Social Determinants of Health (SDOH) greatly influence health outcomes. SDOH surveys, such as the Assessing Circumstances & Offering Resources for Needs (ACORN) survey, have been developed to screen for SDOH in Veterans. The purpose of this study is to determine the terminological representation of the ACORN survey, to aid in natural language processing (NLP).

**Methods:**

Each ACORN survey question was read to determine its concepts. Next, Solor was searched for each of the concepts and for the appropriate attributes. If no attributes or concepts existed, they were proposed. Then, each question’s concepts and attributes were arranged into subject-relation-object triples.

**Results:**

Eleven unique attributes and 18 unique concepts were proposed. These results demonstrate a gap in representing SDOH with terminologies. We believe that using these new concepts and relations will improve NLP, and thus, the care provided to Veterans.

## Introduction

There has been an increased interest in Social Determinants of Health (SDOHs) for over two decades [1]. This is due to the fact that they greatly influence health outcomes and healthcare utilization, thus, contributing to health disparities for disadvantaged individuals [2]. As noted by Powell (2019), SDOHs affect health, behavioral health, and general quality of life [3].

SDOHs are the conditions in which individuals are born, grow, live, work, and age [3,4]. These SDOHs occur across dimensions of functioning, such as social, economic, and physical dimensions [3]. They also occur in various environments and settings, including schools, places of employment, religious centers, and neighborhoods [3]. Examples of SDOH include: (1) opportunities for education and employment, (2) level of income, (3) access to housing and affordable utilities, (4) social and community support, and (5) access to transportation, just to name a few [5,6].

## Screening tools for SDOH

Various tools can be used to screen individuals for SDOH. These include, but are not limited to: WellRx [7]; Protocol for Responding to and Assessing Patient Assets, Risks, and Experiences (PRAPARE) [8]; and Assessing Circumstances & Offering Resources for Needs (ACORN) [9,10]. The ACORN survey is a relatively new tool for measuring SDOH. As such, little is known about the terminological representation of the questions in this survey. Terminological representation is important as it allows for such activities as Natural Language Processing (NLP) and Clinical Decision Support (CDS).

The aim of this research study is to: (1) represent the questions of the ACORN survey using concepts and attributes from the Solor terminology; (2) propose new concepts and new attributes to represent questions of the ACORN survey in cases where needed concepts and attributes are not present in the Solor terminology; and (3) create subject-attribute-object triples using Solor concepts, Solor attributes, proposed concepts, and proposed attributes that represent the ACORN survey questions. This survey has been chosen, as it characterizes Veteran-specific SDOH issues, which currently have not been well represented by terminologies needed for NLP. NLP is important, as it can influence activities downstream, such as CDS. First, we turn to the ACORN screening tool.

## The ACORN screening tool

One tool for measuring SDOH is the Accessing Circumstances & Offering Resources for Needs (ACORN) survey. In 2020, a 13-question survey to screen for SDOH was developed by the Veterans Health Administration (VHA) for use with Veterans [9,10]. This survey uses one question from the WellRx tool, one question from the PRAPARE tool, and five questions from other sources. The remaining six questions were developed by the VHA. Veteran-specific topics on the ACORN survey include: (1) needing information about educational benefits for Veterans, and (2) setting up a video visit with a member of the VA care team [9,10]. Topics not specific to Veterans and not from other sources include: (1) legal issues, (2) having access to and being able to use a smartphone or a computer, and (3) having access to reliable and affordable Internet [9,10].

## WellRx screening tool

As previously stated, one question from the WellRx screening tool was used in the ACORN survey. In 2014, there was no widely available structured method intended or tested for healthcare providers to identify and capture SDOH in the outpatient primary care medicine setting [7]. WellRx, an 11-question screening tool for SDOH, was developed and piloted at the University of New Mexico for this purpose [7]. The questions encompass such topics as: (1) food insecurity, (2) access to housing, (3) affordability of utilities, (4) transportation, (5) employment, (6) education, and (7) safety [7].

## The PRAPARE screening tool

As noted above, one question from the PRAPARE screening tool was used in the ACORN survey. The PRAPARE survey is a 21-question screening tool for SDOH [11]. In 2013, the National Association of Community Health Centers and partners launched a project to develop and implement a national standardized patient SDOH risk assessment protocol, PRAPARE [8]. With its implementation in 2016, PRAPARE provided a way to assess SDOH and expedite actions at the individual, community, and health system levels [8]. PRAPARE covers most of the same topics as WellRx namely: (1) food security, (2) access to housing and utilities, (3) transportation, (4) employment, and (5) education [8]. In addition, PREPARE includes: (1) social and emotional health; (2) being insured or uninsured; (3) clothing needs; and (4) income, just to name a few [8].

## Other sources

As previously mentioned, five ACORN screening tool questions come from other sources. One of these questions, which asks about housing security, originates from clinical reminders from the Electronic Health Record (EHR) used by the VHA [9,10].

The second question from other sources inquires about food insecurity [9,10]. Hager and colleagues (2010) developed and validated a 2-item screening tool measuring food insecurity in families with young children [12]. The question “within the past 12 months we worried whether our food would run out before we got money to buy more” was chosen and modified to be used in order to evaluate food insecurity in Veterans [9,10].

The third question, originating from another source, examines whether or not the Veteran has been notified of the possible shutoff of utilities or water [9,10]. This question was chosen from a 4-question Household Energy Security indicator, and modified for the ACORN SDOH tool [9,10]. This indicator was developed to classify the ability of the members of a household to obtain the energy needed to heat/cool their home and to operate lighting, refrigeration, and appliances [13].

The fourth question developed from other sources identifies how often a Veteran feels lonely or isolated [9,10]. Anderson and Thayer (2018) note that questions from the 20-item UCLA Loneliness Scale measure both feeling lonely and feeling isolated [14]. This ACORN survey question originates from the UCLA Loneliness Scale [9,10].

The fifth, and final, question from other sources assesses how often a Veteran is physically hurt or threatened with harm by someone close to him or her [9,10]. According to Sherin and colleagues (1998), domestic violence is an important issue [15]. Thus, they developed a 4-item scale (HITS) to be used in the family practice setting [15]. The scale represents how often one’s partner is: physically hurt; Insulted; threatened with harm; and screamed at them [15]. The physically hurt and the threatened with harm items have been combined into a question for the ACORN SDOH screening tool [9,10].

## Representing the screening tools

The use of these various screening tools for SDOH produces a wealth of data. These data are a valuable source of health information but currently are not fully utilized by many clinicians [16]. In fact, knowing that a patient has trouble finding transportation, has a potentially unsafe relationship with someone close, is currently unemployed, or various other SDOHs would assist healthcare providers in designing treatment plans to best help the patient [16]. Watkins and colleagues (2020) point out the need for standardized SDOH for care delivery supported by electronic health records: “these SDOH must be gathered, represented, and stored in a standardized way before they can be leveraged by informatics tools designed for health providers” [16]. Terminologies, such as the Systematized Nomenclature of Medicine Clinical Terms (SNOMED CT) and Logical Observation Identifiers Names and Codes (LOINC) can be used to represent these SDOH screening tools and their resulting data.

Arons and colleagues (2018) performed preliminary work to determine how well concepts from six SDOH tools were covered in SNOMED CT, LOINC, ICD-10, and CPT [6]. They noted that although a large number of concepts from these SDOH tools are covered by standardized vocabularies, there exist some gaps [6]. Not surprisingly, Arons and colleagues (2018) demonstrated that the Education, Employment, Housing, Safety, and Social Connections/Isolation domains had particularly high numbers of codes, as these are well covered in SNOMED CT and LOINC [6]. However, domains such as child care, clothing, incarceration, immigration/migration, and Veteran status were found to be lacking codes [6].

The ACORN survey was created two years after Arons and colleagues published their work. Thus, ACORN could not be included in their analysis. It is also possible that additional concepts were added to any or all of the terminologies contained within the Solor terminology within the ensuing years. Therefore, the recent creation of the ACORN survey and the possibility of newly added SDOH-related concepts to SNOMED CT and LOINC provided the impetus for this research.

## The Solor terminology

Solor [17] is an integrated terminology system created in collaboration with the U.S. Dept. of Veterans Affairs (VA) that combines SNOMED CT (representing diseases, findings, and procedures), LOINC (representing laboratory test results), and RxNorm (representing medications) [18]. Solor has two fundamental building blocks: concepts with their synonyms, and semantics [18]. In this case, a concept is a medically related idea, such as heart attack, while a semantic is data that provides contextual meaning to the concepts [18,19]. Like SNOMED CT, Solor is built on a logic model [18]. Most of the concepts are shared by Solor and SNOMED CT and are arranged into hierarchies using “is_a” relationships [18]. Therefore, the modeling is based on SNOMED CT, LOINC, and RxNorm.

As an integrated terminology system, Solor provides many advantages. For instance, this single consistent method of encoding clinical data can allow this data to flow among clinical documentation, decision support applications, and order entry at the point of care [18]. Solor can also support research, quality measurement, and other secondary uses [18].

At the current time, the Solor terminology is used in three different contexts. As noted by Resnick and colleagues (2021) it is used in a research setting [18]. Solor also provides CDS modeling at the VA. In the third context, Solor is part of the Sentinel initiative at the Food and Drug Administration (FDA). Sentinel is the FDA’s national electronic system, which allows researchers to monitor the safety of FDA-regulated medical products, such as drugs, vaccines, biologics, and medical devices [20]. The Sentinel Initiative leverages organizational partnerships in informatics, data science (using natural language processing and machine learning), and other areas [20].

## Methods

The September 2021 version of the Assessing Circumstances & Offering Resources for Needs (ACORN) survey was obtained [9,10]. Each survey question was read to discern all concepts.

Next, Solor was searched for each of the identified ACORN concepts. For those ACORN concepts found to be present in Solor, the codes and names were noted. If the needed concept was not present in Solor, a “new” concept was proposed. This concept most closely represented the meaning of the question and was formed without consulting any other terminology or ontology.

In the final step, Solor searched for appropriate attributes in order to form subject-attribute-object triples. If no appropriate attributes existed, they were proposed. The proposed attributes allowed the existing Solor concept(s) and/or proposed concept(s) representing the question to be connected to the answer choices for that question.

The process of proposing and assigning concepts and attributes was manually performed by author MR. Author PE reviewed the results for consistency. Any disagreement was discussed and a consensus was reached.

## Results

A total of 52 concepts relating specifically to SDOH were identified from the ACORN survey questions. During the encoding process, 12 unique attributes were used: 1 unapproved SNOMED CT attribute and 11 newly proposed attributes (see Table 1).

**Table 1.** Twelve unique attributes utilized

Unapproved SNOMED CT attribute	Proposed attribute
without (unapproved attribute)	
	experienced by has access to has behavior has desire has frequency has lived as has lived in has lived with has need for has truth value scheduled for

A total of 34 unique concepts were used from the Solor terminologies. Of these, 20 were from SNOMED CT; 14 were from LOINC; and 0 were from RxNorm.

As seen in Table 2, 18 unique new SDOH concepts were proposed.

**Table 2.** Eighteen unique concepts proposed

Concept proposed
Affordable internet at home
Apartment/House/Room (no government subsidy)
Apartment/House/Room (with government subsidy)
As a part of household
Cell phone
Friend
Help getting food this week
Help learning to use computer
Help learning to use smartphone
Information about Veterans educational benefits
I do not want internet access at home
Information about Veterans educational resources
Reliable internet at home
Rented housing
Runs out of phone minutes
Smartphone
Steady place to stay
Worry about housing in near future

In Table 3, two of the ACORN survey questions with their triples are shown. As shown in column 2, the subject of the triples is represented by concepts from SNOMED CT or LOINC. The same is true for the object of the triples, as seen in column 4. For these two survey questions, none of the proposed concepts, and three of the 11 proposed attributes were used to form the triples.

**Table 3.** Two Assessing Circumstances & Offering Resources for Needs (ACORN) questions with encodings and triples

ACORN survey question	Subject (SNOMED/LOINC)	Attribute (NEW [from Table1])	Object (SNOMED/LOINC)
(6) How often do you feel lonely or isolated from those around you? a. Often b. Sometimes c. Never	[SNOMED] 267076002 feeling lonely (finding)	“experienced_by”	[SNOMED] 116154003 patient (person)
	[SNOMED ]307048004 feeling isolated (finding)	“experienced_by”	[SNOMED] 116154003 patient (person)
	[SNOMED] 267076002 feeling lonely (finding)	“has_frequency”	[LOINC] LA10044-8 often
	[SNOMED] 267076002 feeling lonely (finding)	“has_frequency”	[LOINC] LA10082-8 sometimes
	[SNOMED] 267076002 feeling lonely (finding)	“has_frequency”	[LOINC] LA6270-8 never
	[SNOMED] 307048004 feeling isolated (finding)	“has_frequency”	[LOINC] LA10044-8 often
	[SNOMED] 307048004 feeling isolated (finding)	“has_frequency”	[LOINC] LA10082-8 sometimes
	[SNOMED] 307048004 feeling isolated (finding)	“has_frequency”	[LOINC] LA6270-8 never
(7) How often does anyone close to you physically hurt you or threaten you with harm? a. Often b. Sometimes c. Never	[SNOMED] 3030701001 person in the family (person)	“has_behavior”	[LOINC] 95,619-3 hurts, insults, threatens, and screams (hits)
	[SNOMED] 394863008 non-family member (person)	“has_behavior”	[LOINC] 95,619-3 hurts, insults, threatens, and screams (hits)
	[LOINC] 95,619-3 hurts, insults, threatens, and screams (hits)	“has_frequency”	[LOINC] LA10044-8 often
	[LOINC] 95,619-3 hurts, insults, threatens, and screams (hits)	“has_frequency”	[LOINC] LA10082-8 sometimes
	[LOINC] 95,619-3 hurts, insults, threatens, and screams (hits)	“has_frequency”	[LOINC] LA6270-8 never

## Discussion

Among the contributions of this work are the triples. These triples can be leveraged with at least two informatics tools: (1) NLP tools, and (2) CDS tools. In the case of NLP, the triples provide increased accuracy in tagging the unstructured text, such as in Electronic Health Record (EHR) data. This can influence other activities downstream.

The High Definition Natural Language Processing (HD-NLP) program is a pipeline developed at the University at Buffalo [18]. The system uses a full semantic parse in memory and then uses an encoder to link text to any set of Ontologies that a user wants to use to represent the knowledge in the free text being codified [18].

As noted above, new concepts and new relations were proposed, and triples were created for each of the ACORN survey questions [21]. Next, the new concepts and relations (attributes) are submitted to be added into the appropriate Solor terminologies (SNOMED CT, LOINC, RxNorm). Once added to Solor, we then can generate triples, where applicable, from the source text using the HD-NLP system. This allows us to identify the SDOH concepts and their relations that exist within the free text of EHR patient records.

Triples can also allow for the triggering of CDS rules. Free text in a physician’s patient note may say, “The patient often feels lonely.” The HD-NLP system would, then, generate the triples: 267076002 feeling lonely experienced_by 116154003 patient; and 267076002 feeling lonely has_frequency LA10044-8 often. From here, these triples would trigger CDS rules, leading to the recommendation of additional services for this patient.

In this way, the use of NLP and CDS can improve the care given to patients. Hence, it is for these reasons that the triples are created while encoding the ACORN survey.

The encoding of the ACORN survey questions revealed three issues: (1) the need to propose new concepts; (2) concepts from more than one terminology that represent any one question; and (3) lack of appropriate attributes or relations. In one of the cases, however, it appeared that SNOMED CT attributes could be used. For example, it seemed possible to utilize “inheres_in” to create the triple: 267076002 feeling lonely (finding) “inheres_in” 116154003 patient (person). However, this is not possible, as SNOMED CT dictates that the domain of “inheres_in” needs to be an observable entity, not a finding [22]. Thus, a new attribute “experienced_by” was proposed.

Almost all of the attributes were proposed (see Table 1). This is most likely due to the fact that the relations or attributes for SDOH are not well represented in SNOMED CT, and thus, Solor. The lack of appropriate attributes demonstrates a gap in the representation of relations between the SDOH concepts.

A second issue involves the representation of the concepts for each question. Many of the questions are represented by concepts from two different Solor terminologies: SNOMED CT, and LOINC (see Table 3). This, in turn, also contributed to the difficulty in finding appropriate attributes or relations to form the triples from these concepts. In other instances, the concepts do, indeed, exist in the same Solor terminology (see Table 3). However, as shown in Table 3, it is still necessary to use a proposed attribute in order to form the triples.

Finally, it was necessary to propose some new concepts (see Table 2). In viewing these concepts, it appears that they represent housing, utilities, and education. Once again, this demonstrates that there is a gap in the coverage of SDOH by the Solor terminologies: SNOMED CT, LOINC, and RxNorm.

Before moving on, a brief note must be made about the lack of RxNorm concepts. This is not necessarily a function of a gap in coverage. Rather, it is most likely due to the content of the questions. In fact, none of the questions ask about specific medications, thus, obviating the need for concepts from this Solor terminology.

There is at least one solution to the previously discussed issue. The proposed attributes and concepts could be submitted for inclusion in SNOMED CT, which would also be included in Solor.

Once this has been accomplished, NLP tools can be used to tag unstructured EHR text with these SDOH concepts from Solor. This will allow us to discover any needs that patients have due to SDOH. From here, healthcare providers can then provide services that their patients need.

After this research was completed, a new September 2022 version of the ACORN survey was published [23]. Only three major changes were made to the newer version.

First, an additional option, “not applicable/I do not pay for utilities,” was added to questions: “(3) How often do you have trouble paying for your utilities (i.e., electric, gas, oil, water, or phone)” and “(3.1) Has the electric, gas, oil, or water company threatened to shut off services in your home [23].” This means that extra triples would be needed for these two questions. The triple 93,670-8 “do you have trouble paying for your gas or electricity bills” has_value LA30226-7 “not applicable” would be used with question (3); and the triple 96,779-4 “has the electric, gas, oil, or water company threatened to shut of services in your home in past 12 months” has_value LA30226-7 “not applicable” would be used with question (3.1).

Second, question “(7) How often does anyone close to you physically hurt you or threaten you with harm” has been removed from the newer version [23]. Despite the fact that the proposed relation “has_behavior” only applies to this question, it will still be submitted for inclusion into the Solor terminologies. It may prove useful, as a similar question may be added to a future version of the ACORN survey. This would mean that free text from a physician’s note in the EHR could appropriately be encoded by the HD-NLP program. Then, these triples, with the “has_behavior” relation, could then trigger CDS rules recommending that healthcare providers obtain additional services for their patients.

Finally, “landline,” has been added to the list of devices for the following question “(10) Do you have access to any of the following devices” [23]. It is likely that some patients served by the VHA only have a landline. In knowing this, healthcare providers can provide health services in a format most appropriate for each patient.

## Conclusion

In conclusion, SDOH are not well represented by the Solor terminologies: SNOMED CT, LOINC, and RxNorm. This gap in representation is especially apparent with the attributes or relations. We believe that by submitting these new concepts and relations to SNOMED CT, and thus to Solor, we can better represent SDOH in these terminologies. From here, NLP programs in conjunction with the improved terminologies can be used with EHR-free text to determine a patient’s need for services due to SDOH, allowing us to provide the best care to our patients.

## Limitations and future directions

One of the limitations of this research is that the newly proposed concepts and relations are most likely applicable only to the ACORN survey. This is due to the fact that they do not appear in the Solor terminologies SNOMED CT and LOINC. Therefore, they may not be generalizable to other SDOH surveys, such as the WellRx and the PRAPARE. For example, concepts, such as, “Information about Veterans educational benefits,” and “Information about Veterans educational resources” are specific to Veterans.

In the future, we will submit the new attributes and concepts to SNOMED CT. In addition, we will use the HD-NLP system with the improved Solor to identify concepts representing SDOH in free-text EHR data. This will allow us to understand which SDOH most affects Veterans. Then, we can provide the services most needed by these patients.

## Supporting information

Resnick et al. supplementary material Resnick et al. supplementary material

## Supplementary material

The supplementary material for this article can be found at https://doi.org/10.1017/cts.2024.24.
