# Supplementary material for: ACORN SDOH survey: Terminological representation for use with NLP and CDS
Source: J Clin Transl Sci. 2024 Feb 6;8(1):e39. doi: 10.1017/cts.2024.24 (PMC10928702; doi:10.1017/cts.2024.24)
Supplement: Resnick et al. supplementary material [file S2059866124000244sup001.pdf]

Assessing Circumstances & Offering Resources for Needs (ACORN) Screening Tool

(1) In the past two months, have you been living in stable housing that you own, rent, or stay in as part of a household?[1]

a. Yes – Living in stable housing

↳ **(1.1) Are you worried or concerned that in the next two months you may NOT have stable housing that you own, rent, or stay in as part of a household?**[1]

i. Yes – worried about housing near future

↳ **(1.2) Where have you lived for MOST of the past two months?**[1]

a. Apartment/House/Room (no government subsidy)
b. Apartment/House/Room (with government subsidy)
c. With Friend/Family
d. Motel/Hotel
e. Short-term Institution like Hospital, Rehab Center, Drug Treatment Center
f. Homeless Shelter
g. Anywhere outside (e.g. Street, Vehicle, Abandoned Building)
h. Other

ii. No – Not worried about housing near future

b. No – Not living in stable housing

↳ ***Collect answer for the question "Where have you lived for MOST of the past two months?"*** [1]

➢ *If respondent endorses either "not living in stable housing" OR "worried about housing near future" for (1):*
(1.3) Are you currently without a place to stay?

a. Yes b. No

(2) I'm going to read you two statements that people have made about their food situation. For each statement, please tell me whether the statement was often true, sometimes true, or never true for your household in the last 12 months.

(2.1) Within the past 12 months, you worried whether your food would run out before you got money to buy more.[2]

a. Often true b. Sometimes true c. Never true

(2.2) Within the past 12 months, the food you bought just didn't last and you didn't have money to get more.[2]

a. Often true b. Sometimes true c. Never true

➢ *If respondent endorses "often true" or "sometimes true" for either "food would run out" OR "food didn't last" for (2):*
(2.3) Do you need help getting food for this week?

a. Yes b. No

(3) How often do you have trouble paying for your utilities (i.e., electric, gas, oil, water, or phone)?[3]

a. Often b. Sometimes c. Never

➢ *If respondent endorses "often"or "sometimes" for (3):*
(3.1) Has the electric, gas, oil, or water company threatened to shut off services in your home?[4]

a. Yes b. No c. Already shut off

(4) How often has lack of transportation kept you from medical appointments, meetings, work, or from getting things needed for daily living?[5]

a. Often b. Sometimes c. Never

➢ *If respondent endorses "often"or "sometimes" for (4):*
(4.1) Do you have an upcoming appointment that you need transportation assistance to?

a. Yes b. No

(Continued)

(5) Do you currently have any legal issues you need help with?

a. Yes b. No

(6) How often do you feel lonely or isolated from those around you?[6, 7]

a. Often b. Sometimes c. Never

(7) How often does anyone close to you physically hurt you or threaten you with harm?[8]

a. Often b. Sometimes c. Never

(8) Do you want help finding or keeping work or a job?[9]

a. Yes, help finding work b. Yes, help keeping work c. I do not need or want help

(9) Do you want more information about educational benefits and resources for Veterans?

a. Yes b. No

(10) Do you have access to any of the following devices? (Please select all that apply.)

- ☐ Simple cell phone (flip phone)
- ☐ Smartphone (a cell phone with a touchscreen and internet)
- ☐ Computer (laptop, desktop, or tablet such as an iPad)
- ☐ None

(11) Do you have access to affordable and reliable internet at home?

a. Yes b. No c. I don't want internet access at home

(12) How often do you run out of phone minutes and/or data before the end of the month?

a. Often
b. Sometimes
c. Never
d. I don't have a cellphone (flip phone or smartphone)

(13) Veterans can now have video visits with their care team from home or another location of their choice. Would you like help setting up a future video visit with a member of your VA care team?

a. Yes
b. No
c. I already know how to do video visits/don't need help
d. I don't know what a video visit is

➢ *If respondent endorses "yes" for (13):*
(13.1) Do you need help learning to use a smartphone, tablet, or computer for video visits at the VA?

a. Yes b. No c. I don't have any of these devices

Sources for questions derived from other screening instruments are listed below, and permissions were obtained for use when needed. When derived from the CMS Accountable Health Communities (AHC) Screener, the original source is cited per AHC guidance. Questions without citations were developed by the VHA ACORN core team and collaborators across multiple VHA offices and sites. For any questions about the ACORN screener, please contact the ACORN team at VHABEDACORN@va.gov.

1. VHA Clinical Reminder
2. Hager, E. R., Quigg, A. M., Black, M. M., Coleman, S. M., Heeren, T., Rose-Jacobs, R., Cook, J. T., Ettinger de Cuba, S. E., Casey, P. H., Chilton, M., Cutts, D. B., Meyers A. F., Frank, D. A. (2010). Development and Validity of a 2-Item Screen to Identify Families at Risk for Food Insecurity. Pediatrics, 126(1), 26-32. doi:10.1542/ peds.2009-3146.
3. Adapted with permission from Page-Reeves J, Kaufman W, Bleecker M, Norris J, McCalmont K, Ianakieva V, Ianakieva D, Kaufman A. Addressing Social Determinants of Health in a Clinic Setting: The WellRx Pilot in Albuquerque, New Mexico. J Am Board Fam Med. 2016 May-Jun;29(3):414-8. doi: 10.3122/jabfm.2016.03.150272. PMID: 27170801.
4. Adapted with permission from Cook, J. T., Frank, D. A., Casey, P. H., Rose-Jacobs, R., Black, M. M., Chilton, M., . . . Cutts, D. B. (2008). A Brief Indicator of Household Energy Security: Associations with Food Security, Child Health, and Child Development in US Infants and Toddlers. Pediatrics, 122(4), 867-875. doi:10.1542/peds.2008-0286.
5. Adapted with permission from the national PRAPARE® social determinants of health protocol developed by the National Association of Community Health Centers, the Association of Asian Pacific Community Health Organizations, and the Oregon Primary Care Organization and their development partners. www.nachc.org/prapare. ©National Association of Community Health Centers. All Rights Reserved.
6. Northwestern University. (2017). PROMIS Item Bank v. 1.0 – Emotional Distress - Anger - Short Form 1.
7. Anderson, G. Oscar and Colette E. Thayer. Loneliness and Social Connections: A National Survey of Adults 45 and Older. Washington, DC: AARP Research, September 2018. https://doi.org/10.26419/res.00246.001
8. Adapted with permission from Sherin, K. M., Sinacore, J. M., Li, X. Q., Zitter, R. E., & Shakil, A. (1998). HITS: a Short Domestic Violence Screening Tool for Use in a Family Practice Setting. Family Medicine, 30(7), 508-512.
9. Identifying and Recommending Screening Questions for the Accountable Health Communities Model (2016, July) Technical Expert Panel discussion conducted at the U.S. Department of Health and Human Services, Centers for Medicare & Medicaid Services, Baltimore, MD.

If you are interested in citing this work, please use the following information:
Cohen AJ, Lehmann LS, Russell LE. "Systematic Screening of Veterans for Health-related Social Needs: An Ethical Imperative." Office of Health Equity/Health Services Research & Development Cyberseminar, U.S. Department of Veterans Affairs, November 2020.

For any questions about the ACORN screener, please contact the ACORN team at VHABEDACORN@va.gov.

Lead Authors
Lauren E. Russell, MPP
Alicia J. Cohen, MD, MSc, FAAFP
Steven Chrzas, MPH
Lisa S. Lehmann, MD, PhD, MSc

Contributing Teams
Veterans Health Administration Office of Health Equity (VHA OHE)
Veterans Health Administration National Social Work Program Office
National Center for Homelessness Among Veterans / Veterans Affairs Greater Los Angeles Healthcare System Homeless Patient Aligned Care Team (NCHAV / VA GLA HPACT) Project Team

Collaborators
Meaghan Kennedy, MD, MPH
Kathleen Mitchell, MPH
Thao Nguyen, MSN, RN
Penni Hernandez, MS, RN
Holly Miller, MS, RN
Leonie Heyworth, MD, MPH
Katie Murray, RN
Diana Santana, MSN, RN
Jay Barrett, RN, BSN, MBA
Robert Craig, RN, BSN
